# Progress, Challenges, and Surprises in Annotating the Human Genome

**DOI:** 10.1146/annurev-genom-121119-083418

**Published:** 2020-05-18

**Authors:** Daniel R. Zerbino, Adam Frankish, Paul Flicek

**Affiliations:** European Molecular Biology Laboratory, European Bioinformatics Institute, Hinxton CB10 1SD, United Kingdom

**Keywords:** human, genome, annotation, genes, variants, regulatory elements

## Abstract

Our understanding of the human genome has continuously expanded since its draft publication in 2001. Over the years, novel assays have allowed us to progressively overlay layers of knowledge above the raw sequence of A’s, T’s, G’s, and C’s. The reference human genome sequence is now a complex knowledge base maintained under the shared stewardship of multiple specialist communities. Its complexity stems from the fact that it is simultaneously a template for transcription, a record of evolution, a vehicle for genetics, and a functional molecule. In short, the human genome serves as a frame of reference at the intersection of a diversity of scientific fields. In recent years, the progressive fall in sequencing costs has given increasing importance to the quality of the human reference genome, as hundreds of thousands of individuals are being sequenced yearly, often for clinical applications. Also, novel sequencing-based assays shed light on novel functions of the genome, especially with respect to gene expression regulation. Keeping the human genome annotation up to date and accurate is therefore an ongoing partnership between reference annotation projects and the greater community worldwide.

## History of the Human Genome and its Annotation

The history of the sequencing and annotation of the human genome is marked by breathtaking acceleration after a prolonged theoretical inception. Many components of the human genome were discovered long before its base pairs were read through astute experimental design.When the DNA was finally readable, these abstract concepts were mapped onto actual sequences, creating a multilayered annotation linking sequence to phenotype.

### Defining Concepts

The concept of genes evolved from theoretical consideration to molecular components (54). In 1866, Gregor Mendel published his laws of genetics (97), and three years later, Friedrich Miescher isolated nucleic acids (31). The term gene itself was coined as early as 1909 by Wilhelm Johannsen (79, 130) to designate the characteristics of the gametes that affect the resulting organism. Even though geneticists did not know the exact molecule involved, statistical analyses of inheritance patterns allowed them to determine that genes were stored in a linear fashion and to start computing genetic maps of gene proximity (143).

It was only in the mid-twentieth century that the experiments of Avery et al. (8) (1944) and Hershey & Chase (65) (1952) demonstrated the role of DNA in carrying genetic information. Once the role of DNA was proven, genes became physical components. Protein-coding genes could be characterized by the genetic code, which was determined in 1965 (109, 135), and could thus be defined by the open reading frames (ORFs). However, exceptions to Francis Crick’s central dogma of genes as blueprints for protein synthesis (30) were already being uncovered: first tRNA (27) and rRNA (87) and then a broad variety of noncoding RNAs (38).

The genome also provides mechanisms to regulate when and where genes are expressed, thus refning their phenotypic effects. In 1939, Conrad Hal Waddington (161) coined the term epigenetics to designate the study of cell type differentiation (67). In 1970, John Gurdon (61) demonstrated that differentiation did not involve changes to DNA, raising the question of how a multicellular organism, whose genome is (nearly) identically replicated across all cells, could express a wide diversity of cell types, tissues, and so on. Epigenetics thus became the study of information conserved across mitosis and not carried by the DNA sequence. Confusingly, the term later came to additionally (and simultaneously) refer to the study of non-Mendelian inheritance across generations (45, 70).

The control mechanism of gene expression levels was illuminated by François Jacob and colleagues through the discovery of the *lac* operon (78), and a model of gene expression regulation was produced: a promoter sequence upstream of the gene to recruit polymerase and operator sequences to recruit transcription factors. Farther away from the promoter, enhancers were found—first in viruses in 1981 (13, 59) and then in eukaryotes in 1983 (9, 55, 98)—to affect transcriptional output at the promoter regardless of distance or orientation.

The genome contains functional regions relevant to its integrity. Centromeric regions, for example, are necessary to recruit the kinetochores to ensure proper separation of chromatin during mitosis, to keep sister chromatids together ahead of mitosis (10), and finally to ensure their own rapid replication during S phase (145). Telomeric regions have long been interpreted to protect the ends of chromosomes, but our understanding of their function is still evolving (133).

Finally, a large amount of the genome is derived from transposable elements. In 1953, Barbara McClintock (95) published the first observation of genes moving in the genome. It was later discovered that transposable elements correspond to repeated sequences that are able to copy themselves within a cell’s genome.

### Reading the Genome

Shortly after the discovery of the importance of nucleic acids, the first (RNA) genome was sequenced in 1976 (47), and sequencing methods were refined to allow for large-scale data production (131). Despite this rapid progress, characterizing the entire sequence of a large genome was still a complex and costly endeavor due to the necessity of collecting genetic linkage maps (101), which meant that the first eukaryotic genome (of yeast) was published only in 1996 (58).

As genome sequencing technology improved, one obvious challenge was to sequence the human genome (155). Despite all the technical obstacles, as early as 1985, scientists such as Robert Sinsheimer at the University of California, Santa Cruz (UCSC), started discussing the feasibility of sequencing the human genome (28). This idea gathered support, and in 1988, a joint project of the US National Institutes and Health and Department of Energy was created to sequence the human genome over a period of 15 years, around which parallel efforts in China, France, Germany, Great Britain, and Japan rallied. The project continued slowly, sequencing less than 15% of the genome over the next 11 years, until the competition of the Celera Corporation created uncertainty about the availability of the sequence and spurred a significant ramping up of resources and processes, leading to the back-to-back release of two draft sequences on June 26, 2000 (76, 157).

### Mapping Old Concepts to the New Sequence

As soon as the sequence of the human genome could be read, the community set out to associate all the earlier concepts inferred through indirect observation onto actual nucleotide sequences and motifs.

#### Gene annotation

Before the sequencing of the entire human genome, fragmentary data were already being collected into reference resources that became the foundation of bioinformatics (140). These were naturally focused on the protein products of genes. In 1965, Margaret Oakley Dayhoff created the first bioinformatic database, the Atlas of Protein Sequence and Structure, and in 1971, Bernstein et al. (15) created the Protein Data Bank (PDB). These resources were followed in the early 1980s by nucleotide sequence databases, such as GenBank and the European Molecular Biology Laboratory (EMBL) Data Library, which would later become EMBL-Bank and then the European Nucleotide Archive. These data collections were enriched by annotation databases such as Swiss-Prot (1986), which later became UniProt, and then domain-specific genomic annotations such as RepBase (1992), AceDB (1995), FlyBase (1997), and WormBase (2001). To collect sequence polymorphisms, the dbSNP reference database was created in 1999 (134), establishing an unambiguous assignment of identifiers to single-nucleotide polymorphisms (SNPs), known as reference SNP IDs (rsIDs).

As soon as the raw genomic sequence was released, various teams competed to annotate the gene loci. The initial methods developed fell into three broad categories. The first consisted of ab initio methods such as GENSCAN (20), which used contemporary biological knowledge of transcription, translation, and splicing to build computational models that looked for signals of these processes in the genomic sequence itself and required no additional input. The second was gene annotation methods such as SGP2 (113), SLAM (3), and TWINSCAN (48), which built computational models that leveraged knowledge of patterns of sequence-level conservation among species to identify protein-coding genes subject to purifying selection. The third was to take experimental data from one or more sources of sequenced cDNA and expressed sequence tag (EST) libraries, held in the International Nucleotide Sequence Database Collaboration (INSDC) databases (81), and curated annotation from expert databases such as Swiss-Prot (now UniProt) (154).

##### Manual and automated annotation

**Figure 1:**
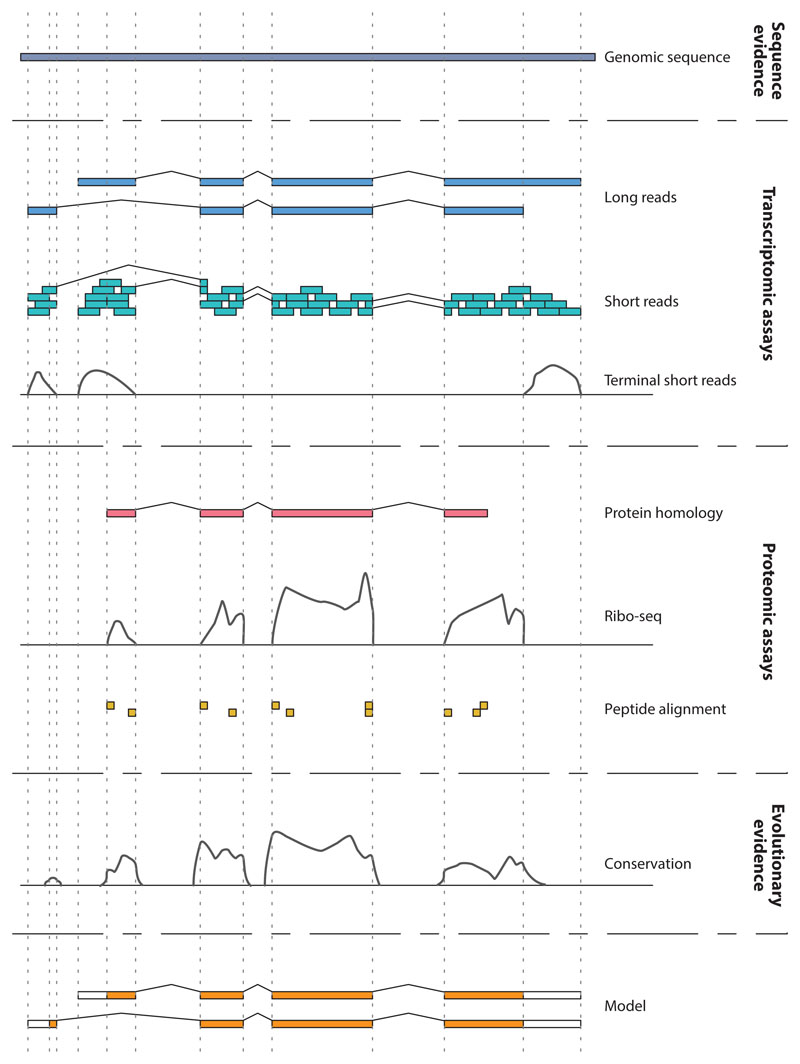
Gene annotation process. Gene annotation uses diverse orthogonal data types to determine first the structure and then the most likely functional class of the transcript and gene locus. Long transcriptomic data aligned to the reference genome identify the overall exon-intron structure of the transcript, while short RNA sequencing reads give confidence to the annotation of precise intron/exon boundaries and extensions at the ends of the transcripts (5′ and 3′ untranslated regions), especially where coverage from longer reads is low. Some transcript structures may be annotated entirely based on RNA sequencing data, again where coverage from longer reads is low. Terminal short-read data sets help define the 5′ and 3′ ends of transcripts, which is important from both a structural and functional point of view; where the termini of a transcript can be identified with confidence, lending certainty of the structural annotation, the annotators gain greater confidence in their determination of functional annotation. The presence of high-quality proteomic data and evidence of the evolutionary conservation of coding sequence informs the annotation of coding potential.

While some evidence-based approaches, such as Ensembl and UCSC genes, were purely computational, both the RefSeq group at the National Center for Biotechnology Information (NCBI) (110) and the Human and Vertebrate Analysis and Annotation (HAVANA) group (49) [initially at the Wellcome Sanger Institute, now merged into Ensembl at EMBL’s European Bioinformatics Institute (EMBL-EBI)] employed manual annotation approaches to complement automated annotation methods. Manual approaches not only require annotators to examine all alignments that are used to create gene and transcript models but also allow them to take into account any orthogonal data, including critical reading of the available literature, to determine the best representation of a gene feature (for a summary, see Figure 1). The manual approach is thus able to give a highly sensitive and specific annotation at the cost of speed. Indeed, the full manual first-pass annotation of the human reference genome by the HAVANA group took approximately 13 years.

In 2006, the human Encyclopedia of DNA Elements (ENCODE) Genome Annotation Assessment Project (EGASP) compared automated annotation pipelines with HAVANA manual annotation of the ENCODE pilot regions, representing 1% of the human genome (40). This study revealed that, while the best automated annotation pipelines were broadly successful in identifying manually annotated protein-coding gene loci, all methods failed to reproduce the manually determined transcript exon–intron structures, particularly where alternatively spliced transcripts were identified (60). Although far more laborious, the manual annotation provides a detailed review of each edge case and the opportunity to select the evidence relevant to each locus. Manually encoding an algorithm to handle each and every exception would be less cost-effective than directly editing these occurrences in a database. It is, however, conceivable that recent developments in machine learning will enable a computer to devise such knowledge automatically, in which case existing manual gene annotations will prove an invaluable training data set.

Notwithstanding the general adoption of these two reference sets for gene annotation, additional approaches to gene annotation continue to be developed. For example, as well as the automated gene annotation methods that use one or two sources of data, methods such as AUGUSTUS (66) and Maker (21) have been developed that integrate multiple sources of data, including other gene predictions and data from RNA sequencing (RNA-seq). Though these approaches could be used to annotate the human genome, their stated role is to support gene annotation for genome projects with substantially less data and attention to annotation than the human genome.

##### Advances in transcriptome sequencing

The emergence of new transcript sequencing technologies has supported new approaches for detecting genes and transcripts along the human genome (see Table 1). The first of these next-generation sequencing technologies, RNA-seq (163), was based on Solexa (12) (now Illumina) sequencing and provided significantly higher depth (i.e., more sequenced molecules) than Sanger cDNA reads but with much shorter reads. While the length of reads for the technology has extended from approximately 30 bases in early versions to a maximum of approximately 250 bases today (and a general practical application of approximately 100 bases), the shorter length of reads compared with INSDC cDNA data hampers their assembly into full-length transcripts, which can be several kilobases long.

This problem was exposed by the RNA-seq Genome Annotation Assessment Project (RGASP) (139), a recapitulation and extension of the EGASP exercise that focused on RNA-seq data. RGASP showed that no method achieved the same level of quality as automated annotation pipelines using Sanger-sequenced INSDC data sets in EGASP. Despite the development of new methods such as StringTie (119) and improvements in the pioneers of RNA-seq transcript assembly such as Cufflinks (151), the fundamental difficulty in assigning short reads to longer transcripts that are subject to alternative splicing with the required resolution appears to be insurmountable (88, 150, 164).

Sequencing technologies generating longer reads, such as Roche’s 454 pyrosequencing (94); Pacific Biosciences’ Single Molecule, Real-Time (SMRT) sequencing (39); and Oxford Nanopore sequencing (99), can aid the reconstruction of transcripts. The latter two methods are still relatively new, but their read length and coverage depth hold the promise of solving the problem of accurately identifying transcript structures. While none of these methods produce reads with the same low error rate as Sanger-sequenced cDNAs, when polished by consensus generation (128), RNA-seq data (146), and variation data (170), they can be used in an equivalent manner for gene annotation by both automated and manual approaches. Combined with intron-spanning RNA-seq reads to validate splice sites with base-pair resolution, they promise to revolutionize transcript annotation in the near future.

##### Protein-coding genes

Protein-coding genes were the best-understood class of gene features prior to the sequencing of the human genome, with the Swiss-Prot and RefSeq databases providing genome-free curation of protein and gene sequences, respectively. Despite this foreknowledge, the total number of protein-coding genes is still being debated (see the sidebar titled First Surprise: How Many Protein-Coding Genes Are There in the Human Genome?). Frequently, short ORFs are found to be transcribed, suggesting the existence of uncharacterized proteins (see the sidebar titled Second Surprise: Lilliput Genes). In some cases, the evidence from nonreference databases aligns to genomic regions that do not contain an intact coding sequence. Such inconsistencies arise either from sequencing errors in the reference sequence or from natural polymorphisms. Genuine loss-of-function variants in the human reference sequence have been identified at a range of allele frequencies, with some gene regions containing very rare alleles on the reference sequence that were initially thought to be nonfunctional pseudogenes. To correct these inconsistencies, the Genome Reference Consortium (GRC) has supplemented many affected regions with patches and representations of alternative alleles to allow the functional copies of protein-coding genes to be captured in the total gene set (132).

##### Pseudogenes

Pseudogenes are predominantly duplicate copies of genomic loci that share sequence similarity with their functional parent copy but lack protein-coding potential due to the presence of disruptive mutations such as frame shifts and premature stop codons. Pseudogenes are classified according to the biological processes that led to their creation as (*a*) processed pseudogenes, which are created by retrotransposition of mRNA from functional protein-coding loci back into the genome, or (*b*) duplicated or unprocessed pseudogenes, which are created by the complete or partial duplication of functional genes; a third and distinct category is (*c*) unitary pseudogenes, which are created by loss-of-function mutations in ancestral functional protein-coding genes (116). Pseudogenes are of interest not only because of the insights they can provide into these processes but also because their shared homology with functional protein-coding parent genes can inform interpretation of the alignment of transcriptomic data to the genome. In addition, pseudogenes are a substrate on which evolution can occasionally act to create novel function; for example, the long noncoding RNA (lncRNA) responsible for X inactivation arose from a duplicated or unprocessed pseudogene (37) (see also the sidebar titled Third Surprise: Win Some, Lose Some).

##### Noncoding RNA

The human genome is pervasively transcribed, with the vast majority of the bases in the reference human genome represented in transcriptomic data sets (34, 41). The resulting transcripts that do not belong to protein-coding genes are usually divided by length (141).

Small-RNA genes are conventionally characterized by the fact that they are shorter than 200 base pairs, do not encode polypeptides, and possess secondary structures that are important to their function. They are generally identified in the genome by (*a*) homology to sequences of known genes both within the same species and between species; (*b*) the presence of a known secondary structure; (*c*) the presence of paired changes in sequence or covariance that preserve structure (105); and, more recently, (*d*) the presence of small-RNA sequences detected experimentally. Small RNAs are often found in large numbers in the human genome; for example, the approximately 2,000 microRNAs generate massive diversity in their targets through sequence differences in the mature microRNAs, while the U6 small nuclear RNAs have more than 1,300 copies of essentially the same sequence. While the numbers of potential genes may be large,many loci encode nonfunctional (or pseudogenic) copies of the small RNA, and discriminating between the functional and nonfunctional copies remains a problem (36, 86). However, the development of computational methods combined with manual curation and literature review of expert small-RNA databases holds the potential to achieve greater resolution of gene classes where the biology is better understood and where experimental data provide sufficiently comprehensive coverage. The functions of many small RNAs have been very well characterized, and both germline and somatic variation have been linked to disease. As such, it is as important to obtain a full representation of functional small-RNA loci in the reference genome as it is for protein-coding genes.

lncRNAs are a class of transcripts that, by definition, are more than 200 bases in length, frequently extending to tens of thousands of kilobases. Unlike small RNAs, lncRNAs lack known RNA secondary structures, although there are considerable ongoing efforts to investigate whether functional and/or structural motifs can be identified and used to inform the annotation and classification of lncRNAs. lncRNAs generally show little cross-species conservation at the sequence level, although they more commonly show conservation of their position in syntenic regions of the genomes.

Large numbers of lncRNA loci have been identified in the reference annotation catalogs (approximately 18,000 in Ensembl/GENCODE and 15,000 in RefSeq). Even larger catalogs have been created by transcript reconstruction for RNA-seq data (69), and resources that collate other individual catalogs reach even greater numbers of lncRNAs—LNCipedia, for example, contains approximately 49,000 high-confidence loci (160)—although different resources have different criteria for annotation, making direct comparison difficult. Given the rate of discovery of new lncRNA loci in both RNA-seq and long transcriptomic data sets, it is unlikely that these figures represent the final tally.

Some lncRNAs have been clearly demonstrated to be functional. The X-inactive specific transcript (*XIST*) locus, for example, is an essential component of the X-inactivation process (122). While only a few lncRNA loci have been characterized to the same depth as *XIST*, more lncRNAs, such as *XIST* and *HOTAIR*, have been implicated in the regulation of epigenetic modifications (129) as well as other processes, such as the regulation of transcription (62). lncRNAs such as *HOTAIR* and *MALAT1* have been implicated in disease (84, 171), and while the mechanism for their involvement is frequently unclear, they may serve as useful markers for prognosis via the monitoring of expression levels (127).

#### Repetitive regions and transposable elements

A large proportion of the human genome consists of repetitive sequences. Transposable elements make up the largest category, covering approximately 45% of the genome, and possess the innate ability to move around the genome (112). The vast majority of transposable elements (approximately 90%) are retrotransposons, which are initially transcribed from DNA to an RNA intermediate before being copied back to DNA by reverse transcriptase enzymes (29). The DNA copy is then inserted back into the genome in a new position, often far from the original locus. Long terminal repeat and long interspersed nuclear element (LINE) retrotransposons encode the reverse transcriptase enzymes that catalyze their creation, but short interspersed nuclear element (SINE) retrotransposons do not. DNA transposons do not utilize an RNA intermediate and instead are excised from the genome and reinserted via the activity of a transposase enzyme. As with the retrotransposons, some classes of DNA transposons encode their own transposases, while others do not and rely on the presence of other transposons for their mobility. The remaining repeat sequences comprise microsatellites, which are very short DNA sequences (typically 5 or fewer bases in length) repeated many times; larger minisatellites (10–60 bases in length); and satellite DNA, such as alpha- and beta-satellite DNA, which forms the main component of centromeres and heterochromatin. Repeat sequences are identified in the genome on the basis of sequence similarity to curated repeat libraries by computational methods such as RepeatMasker (148; http://www.repeatmasker.org).

#### Polymorphisms

SNPs are characterized by their alleles and the shared flanking sequences, and mapping them to the genome is therefore a matter of performing a sequence search in the genome (24). Since the human reference genome is composed of sequences from a few donors, largely from the anonymous RPCI-11 donor (111), the scientific community endeavored to enrich it with common polymorphisms sampled across wide populations. In some cases, the GRC has added the sequences of alternative haplotypes for highly variable regions of the genome, such as the major histocompatibility complex and leukocyte receptor cluster. Large surveys such as the International HapMap Project (75) and the 1000 Genomes Project (1) have further enriched our knowledge of the genome with short polymorphisms as well as structural variants. These maps have provided researchers with allele frequencies across populations as well as linkage information. Once they are annotated onto the genome, interpreting the functional impact of variants is very much an open research question; however, this process is sensitive to the reference annotations used for genes, regulatory features, repeats, and so on (82). This increasing reliance on annotations for biomedical applications in particular is a driver for current efforts to ensure that annotations are both complete and stable.

## GRCH38: The Human Genome and its Current State of Annotations

**Figure 2:**
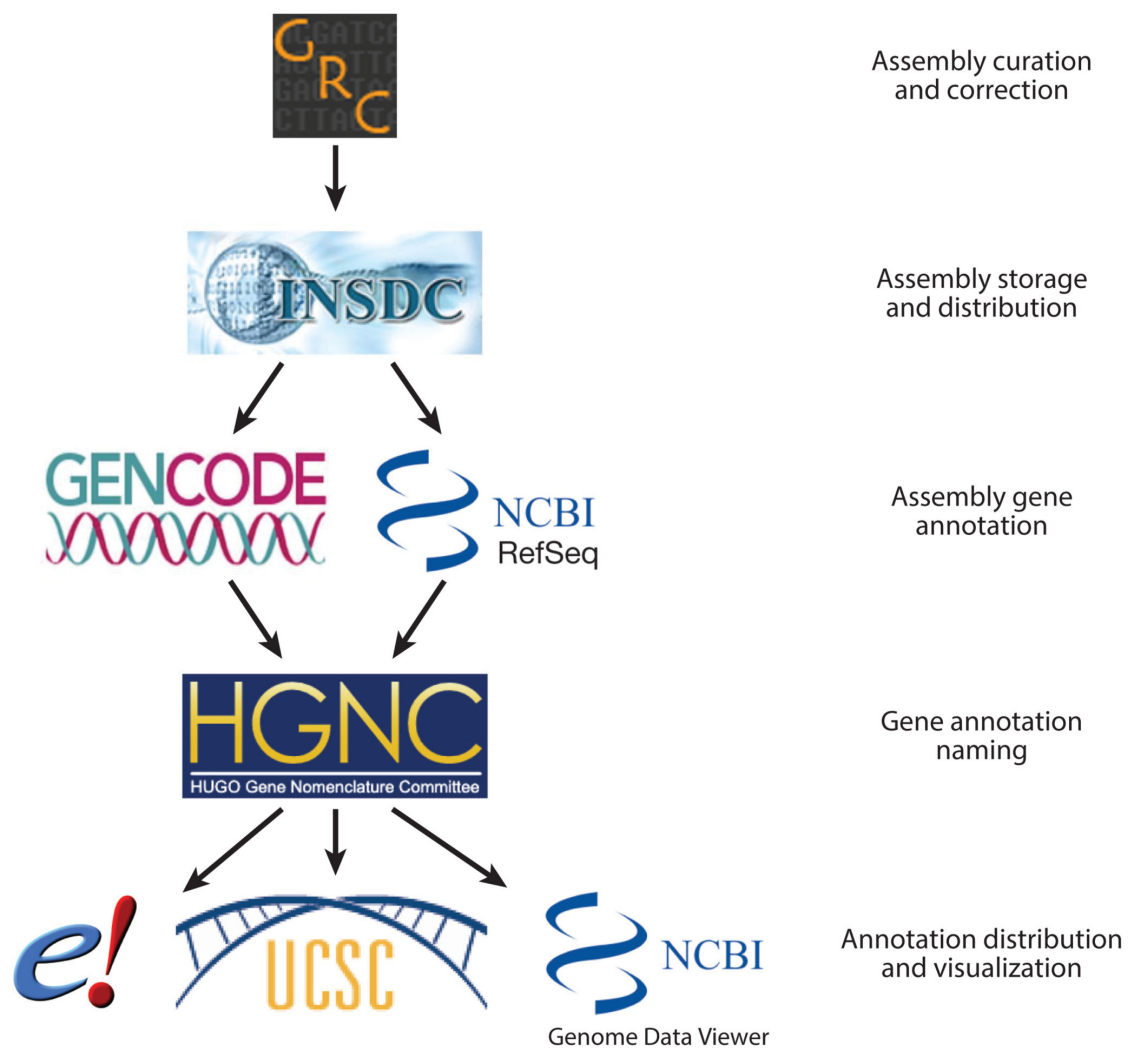
Organizations that support the GRC assembly and its gene annotations. Abbreviations: *e!*, Ensembl Project; GRC, Genome Reference Consortium; HGNC, Human Genome Organisation (HUGO) Gene Nomenclature Committee; INSDC, International Nucleotide Sequence Database Collaboration; NCBI, National Center for Biotechnology Information; UCSC, University of California, Santa Cruz.

The current official GRCh38 genome assembly and its annotations are a corpus of public knowledge that is kept up to date and accurate under the stewardship of multiple specialist bodies across the world, as illustrated in Figure 2. The GRC (25), a collaboration among five institutes, defines the official genome build sequence and hence lends its name to the assembly. It is responsible for improving the human reference genome assembly, correcting errors, and adding sequences to ensure that it provides the best representation of the human genome to meet basic and clinical research needs. Every time a release or an update is ready, it submits the sequence to the INSDC (81), which freely distributes the sequence via three international nucleotide archives in Japan, Europe, and the United States.

Once the raw sequence is available, it is vital to assign known elements to it, so that past research, mapped to a previous genome assembly, is not rendered meaningless by a change in the coordinates. This process ensures the backward compatibility of the new build with past research. Human genes are annotated in parallel by two consortia: GENCODE (49) and RefSeq (110). This two-pronged effort serves to stimulate research by providing a point of comparison. These two annotations are regularly compared, producing the Consensus Coding Sequence (CCDS) annotation (125). To mitigate the confusion that could be created by the use of two different reference annotations, the Human Genome Organisation (HUGO) Gene Nomenclature Committee (HGNC) (18) is responsible for assigning common gene names and symbols to both annotations. Variants are separately mapped to the genome by dbSNP (131) and Ensembl (172). All of these annotations are then freely distributed via public genomic databases and browsers, particularly Ensembl, the UCSC Genome Browser (63), and the NCBI Map Viewer (167).

An accurate representation of the gene content of the human genome is of great importance both for supporting research in genome biology and as a foundation for the interpretation of genetic variation in the clinic. Given the relative inaccuracy of even the best automated methods and the chance (or even likelihood) that any error in gene annotation could be propagated into an error in the clinic, the two sets of gene annotations that are generally utilized as a reference are predominantly manually created and maintained on one hand by the Ensembl group in collaboration with the GENCODE consortium (formally known as the Ensembl/GENCODE annotation) and on the other by the RefSeq group.

## GRCH39 and Beyond: Future Challenges of Human Genome Annotation

The concept of the reference human genome is changing with the creation of the Human Pangenome Reference Consortium (https://humanpangenome.org), which plans to complete several hundred high-quality haplotype-resolved human assemblies representing populations around the world. These genomes will be collected and presented in a graph-based pangenome structure to best represent human genetic variation. The pangenome and extracts of it representing individual human genomes will be the substrate for future genome annotation and analysis.

### The Genome as a Template for Transcription

Despite tremendous progress since the publication of the draft genome, the identification and characterization of transcribed regions of the genome are still moving targets, as we learn more about the subtleties of transcriptions. Thus, annotations are continuously being enriched with subtle new features revealed through novel assays.

#### Converging on a final list of protein-coding genes

**Figure 3:**
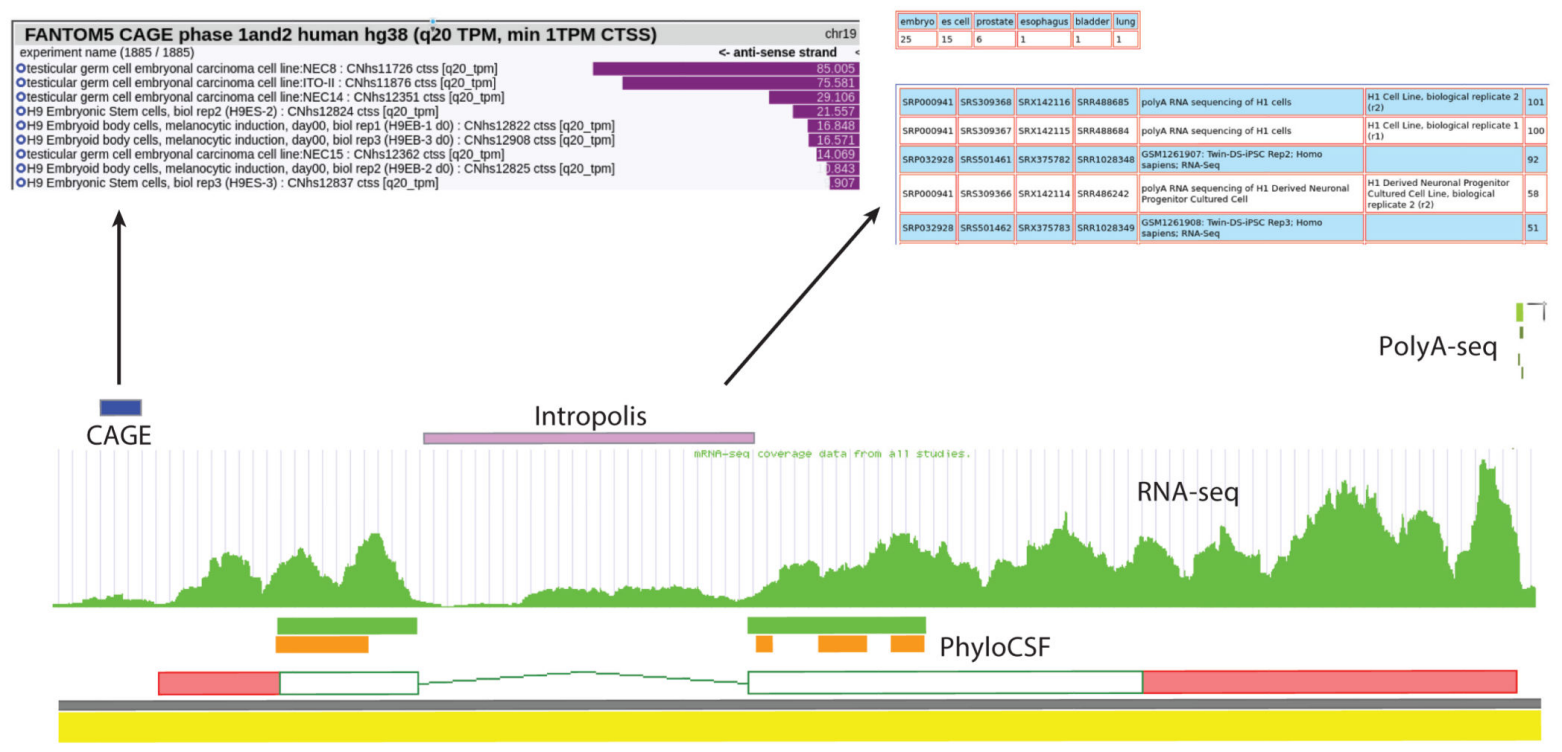
A locus whose identification was possible only through the analysis of recent orthologous data types. The locus lacks any support from transcript evidence deposited in INSDC databases, and as such, it is not represented in any reference annotation database. Only by identifying the intersection of PhyloCSF data (to identify conserved protein-coding potential), RNA-seq data (to provide evidence of transcription and tissue specificity), Intropolis RNA-seq-supported intron-spanning reads (to provide evidence for precise split junctions and support tissue specificity from other datasets), CAGE data (to define transcript 5′ ends and tissue specificity support), and polyA-seq data (to define transcript 3′ ends and tissue specificity support) could a correctly splicing transcript model be built and the correct coding sequence added. Given the expectation of conservation, protein-coding genes identified by this annotation process were also annotated in mouse to provide an additional check on their validity Abbreviations: CAGE, cap analysis gene expression; INSDC, International Nucleotide Sequence Database Collaboration; PhyloCSF, Phylogenetic Codon Substitution Frequencies; polyA-seq, polyA sequencing; RNA-seq, RNA sequencing.

**Figure 4:**
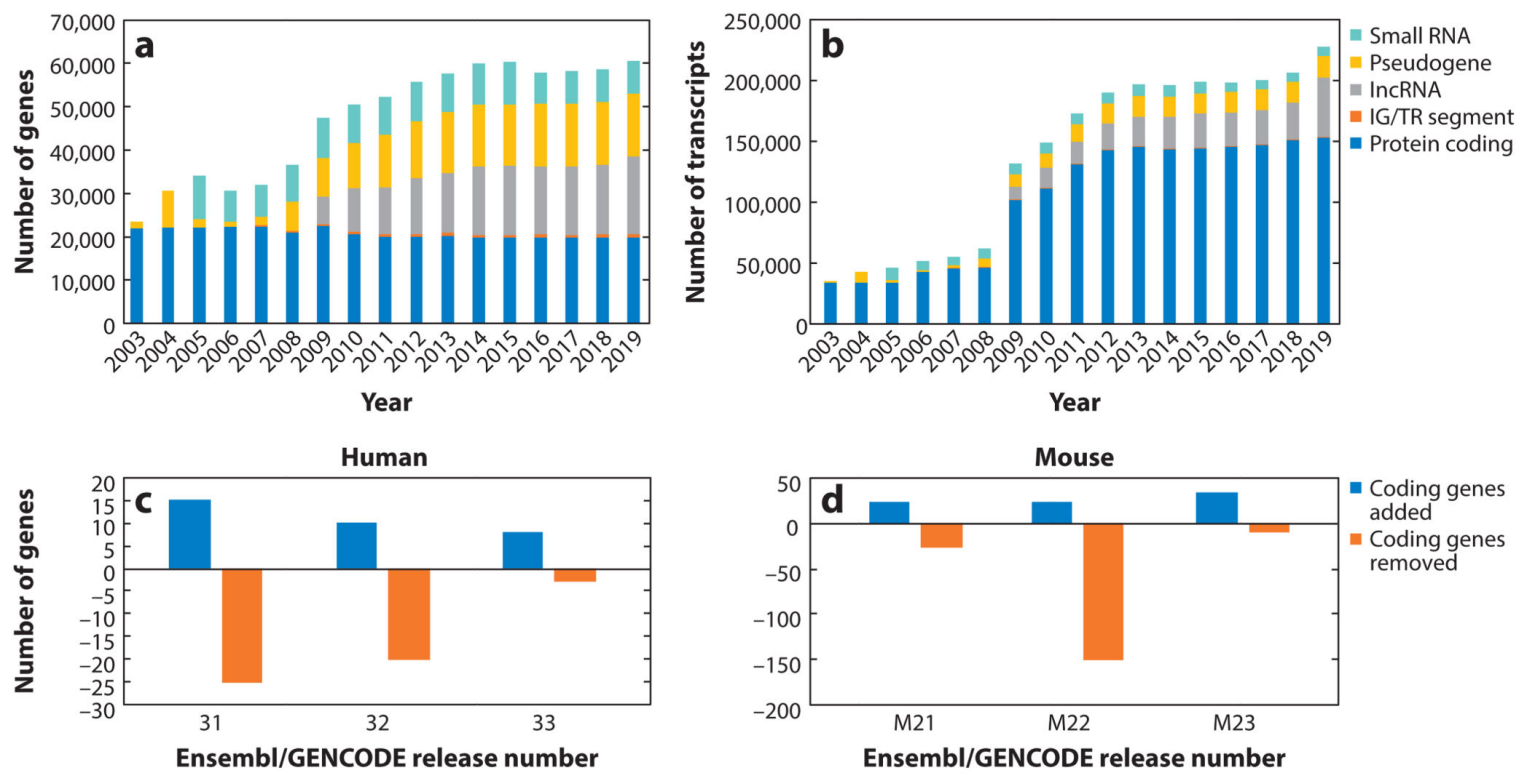
Progress in the annotation of gene loci in Ensembl/GENCODE. (*a*) The number of protein-coding genes annotated has generally fallen over time but appears to be generally stable in recent years. The number of pseudogene loci increased rapidly during the annotation of the whole genome (2007–2012) and has maintained slow growth subsequently, while the number of lncRNA experienced a similar pattern of increase but continues to rise. Small-RNA locus totals are generally stable, only changing when there is a significant update to their automated annotation pipeline, and the relatively few IG and TR segments have remained broadly stale since their initial annotation. (*b*) The number of transcripts continues to increase over time, particularly for protein-coding genes and lncRNA loci, and given the availability of high-quality long-read data sets, this trend is expected to continue. (*c*,*d*) The changes to protein-coding gene counts underlying the relatively stable headline totals for human and mouse, respectively, in three recent Ensembl/GENCODE annotation releases. Protein-coding genes were both added and removed in every human and mouse release, with a total of 33 additions and 48 removals in human and 80 additions and 188 removals in mouse, suggesting that the final gene annotation for protein-coding genes has not yet been settled. Abbreviations: IG, immunoglobulin; lncRNA, long noncoding RNA; TR, T cell receptor.

New genes are being regularly detected thanks to a combination of better computational methods to generate and rank targeted lists for manual review (92) and a growing and diverse corpus of transcriptomic and proteomic data sets that cover an expanding number of human cell types and tissues (for an example, see Figure 3), experimental resources also employed by gene annotation resources such as the Comprehensive Human Expressed Sequences (CHESS) catalog (120). While the use of such resources is clearly of tremendous importance in the discovery of new protein-coding genes, the total number of protein-coding genes in reference catalogs is converging on stability, as illustrated in Figure 4. At the same time, many protein-coding annotations are being removed as well. For the most part, this removal happens as an older annotation is reevaluated in the light of better functional, evolutionary, transcriptomic, proteomic, and human variation data on a case-by-case basis. When a locus that was previously annotated as protein coding is found on review to lack the expected level of evidence for a protein-coding gene, its classification will be updated (44).

#### Converging on a definition of protein-coding genes

Given the clear benefit of removing uncertainty from the annotation of protein-coding genes in the reference genome, significant efforts have been made to achieve convergence among the major reference databases, such as the CCDS project being carried out by RefSeq, Ensembl/GENCODE, UCSC, UniProt, and the HGNC (126). While these cross-database exercises have made great strides toward achieving the goal of convergence, they have also revealed some of the remaining gaps in our knowledge, particularly questions on the very definition of a protein-coding gene.

Specifically, new evidence has shown low-level transcription and translation across the genome, although this may not have a role in cellular physiology. The depth of transcriptomic data available allows us to identify a greater number of transcribed regions of the genome. At the same time, new techniques such as ribosome profiling (ribo-seq) provide direct evidence of translation (via the proxy of interaction between ribosome and transcript), demonstrating that translation is perhaps more promiscuous than previously thought (72) (see also the sidebar titled Fourth Surprise: Coding Noncoding RNA?).

Additional methods are therefore required to discriminate functional protein-coding loci from other transcribed and translated regions. Current approaches rely on better determination of evolutionary conservation to provide additional confidence in protein-coding potential, but this precludes the annotation of genuinely emergent functional coding genes (80, 144). A similar class of putative protein-coding genes is those that have clear evidence of transcription, and sometimes translation as well, but have activity restricted exclusively or predominantly to a disease state. Cancer–testis (CT) antigen genes such as GAUGE family members display these characteristics of protein-coding genes but lack evolutionary conservation, and we have no understanding of the role they play in normal cellular function. They are potentially important targets for immunotherapy (57) and demand inclusion in the reference gene catalogs; however, their existence suggests that further subclassification of protein-coding genes is required to capture the functional diversity within the group.

A relatively small number of protein-coding genes have been thoroughly investigated in direct experimental assays to establish their function, although approximately 87% have been detected in high-confidence proteomic experiments (53) (see also the sidebar titled Second Surprise: Lilliput Genes). As such, the determination of protein-coding potential still requires identifying signals of purifying selection on the coding sequence of protein-coding genes (92). While this is partly due to the lack of available primary data—for example, from embryonic or developmental tissue, or subregions of organs such as the brain—other approaches are needed to validate at least the protein-coding potential of a locus, if not its function. One such approach is to raise antibodies against all putative protein-coding loci and use them to detect proteins in a variety of assays, including western blots and immunohistochemistry (153). The latter can be useful in giving hints to function via determination of tissue and subcellular localization. Furthermore, the generation of the antibody itself creates a reagent that can be used in other assays, such as coimmunoprecipitation to identify protein–protein interactions. Similarly, large-scale testing of protein–protein interactions via assays, such as yeast two-hybrid systems, can also provide additional validation for the functional potential of a coding locus.

#### Transcript annotation

Virtually all protein-coding gene loci are alternatively spliced, meaning that they are transcribed into a variety of transcripts that each include only a subset of the ex-ons at their locus (64, 106). There is frequently disagreement on whether some or all transcript isoforms of a locus are biologically relevant (17) or only one is important (152). One view is that almost all alternative splicing is created by stochastic events during transcription and splicing, creating biologically inert transcripts that could be considered noise (96). Relatively few alternatively spliced transcripts have been functionally characterized. Historically, several approaches have been used to quantify the expression levels of transcripts within a gene, including reverse transcription PCR, ESTs, and microarrays, but RNA-seq has much greater throughput than reverse transcription PCR and ESTs and outperforms microarrays in its throughput, sensitivity, identification of DNA variation, dynamic range, and lack of reliance on existing annotation (174). However, long transcriptomic data may now be used for quantification (149, 169) and may overtake RNA-seq in quantification for the same reasons of length and connectivity that will enable it to do so in transcript annotation.

RNA-seq quantification of individual transcripts suggests that some are persistently more highly expressed than others. However, function has been demonstrated in alternatively spliced transcripts that have long been dismissed as noise, such as isoforms that retain intronic sequence even in their mature forms and those predicted to be targeted by nonsense-mediated decay (NMD) (see the sidebar titled Fifth Surprise: Nonsense-Mediated Decay—Transcription’s Autocorrect). It must be acknowledged that we currently lack the biological understanding to accurately determine whether an individual transcript is functional and, if so, what its function is. However, in the absence of experimental characterization, features such as evolutionary conservation and a high expression level of alternatively spliced transcripts can be strong indicators of functional potential, and a lack of conservation and low expression suggest the opposite. However, while transcripts that do not display these features may be functional either by encoding an alternative protein or by having a regulatory effect, some transcripts may not be functionally important in their own right or even in the context of contributing to or buffering the overall transcriptional output of the gene. Annotation should accept this and seek to add information regarding function and proxies to function (both positive and negative) to transcripts as it emerges.

#### Read-through genes

Read-through or chimeric transcripts share exonic overlap with two or more loci on the same strand (56). These transcripts were first identified in INSDC data, but the increased sequencing depths of second- and third-generation sequencing technologies (102, 124) may make them more readily identifiable, particularly where genes lie close to one another on the same strand. While some read-through transcripts contain ORFs that span the coding sequence of all the loci they overlap, many others do not and are predicted to be subject to NMD. Read-through transcripts tend to be weakly expressed relative to the protein-coding loci they connect, and although they are clearly not technical sequencing artifacts, it remains unclear what functional role they play (if any) in either increasing protein diversity or regulating the expression of the loci they overlap.

#### Defining long noncoding genes

One of the difficulties for the annotation or description of genomic features in a world where long and deep transcriptomic data sets are readily available is the increase in the identification of novel transcripts that connect previously independent gene loci. For protein-coding genes where the functional region (the coding sequence) is readily identifiable, this presents less of a problem and can be mitigated by the identification and tagging of transcripts that read through between more than one locus. For long noncoding RNA genes, it is much more of a problem; their functionality is less well understood, both in general terms and regarding which parts of these transcripts are functional effectors. Thus, if novel transcripts connect two annotated loci, it is hard to determine whether the true locus was originally fragmented or whether merging them together is wrong. Incorrectly merging annotations has downstream ramifications for analyses such as locus-level expression quantification. This problem is also compounded in lncRNAs by their generally low and tissue-specific expression, which means that it is often difficult to use orthogonal data such as expression levels derived from RNA-seq to join or break apart loci.

### The Genome as a Vehicle of Genetics

From initial surveys of polymorphisms across the general population, targeted projects are now attempting to annotate the functional relevance of variants, especially in a medical context. Thus, large patient cohorts were consented for research by projects such as the International Cancer Genome Consortium (74), the Cancer Genome Atlas (104), Pan-Cancer Analysis of Whole Genomes (22), and the UK Biobank (4). In some cases, this sample collection is integrated into patient care strategies, as in Genomics England and other national initiatives (138). These studies can be analyzed via an array of approaches, ranging from genome-wide association studies, as stored in the NHGRI-EBI GWAS Catalog (19) for common diseases, to individual and familial case studies for rare variants, such as Online Mendelian Inheritance in Man (OMIM) (5), ClinVar (89), ClinGen (35), Orphanet (115), and Deciphering Developmental Disorders (33).

When scaling up to cohorts of millions of patients, it becomes increasingly important to eliminate even occasional artifacts to reduce false positive discoveries. For example, one avoidable source of bias occurs when mapping short sequencing reads to the haploid reference genome. Indeed, reads with the alternate allele of a variant map fractionally less often than reads of the same genomic location with the reference allele. To eliminate this bias, new bioinformatic tools use graph structures to map these short reads to an augmented graph genome that contains the reference as well as all known variants (114). It is likely that, in the future, the human reference genome will be such a graph genome.

Storing the genome as a graph would also cleanly resolve the issue of annotating the segments of immunoglobulin and T cell receptor genes, which is problematic even for the International Immunogenetics specialist reference database (91). These loci are brought together during V(D)J recombination in developing lymphocytes during B and T cell maturation. As a result of this combinatorial operation, there is significant structural variation among the individual lymphocytes within an individual, and it is therefore difficult to provide a meaningful consensus annotation of that region.

### The Genome as a Functional Molecule

The last frontier of genomic annotation remains the gene regulatory system, as this system is necessary for the expression of a gene and could even be included in the definition of the gene (54). When considered as a molecule, the genome has many dynamic yet reproducible characteristics that can be assayed (50). From the larger to the smaller scale, it is possible to measure, for example, chromatin loops, chromatin accessibility, histone marks, transcription factor binding, shape, and DNA modifications.As with gene expression,detecting patterns requires assaying these properties across a large number of tissues, cell types, and conditions; hence, large consortia such as the Roadmap Epigenomics Mapping Consortium (14), ENCODE (41), and BLUEPRINT (2), brought together with others under the umbrella of the International Human Epigenome Consortium (142), are currently collecting a substantial array of tissue- or cell type–specific measurements. This multiomic approach to functional genomics was dubbed epigenomics (not to be confused with the already overloaded term of epigenetics). In parallel, several assays can test for regulatory effects using either natural (108) or engineered (117) sequence variation.

Despite the plethora of assays and measurements, converting the classical definition of enhancers into genomic or epigenomic terms is still a matter of intense discussion (52, 83, 118, 136, 159), as no strong distinguishing pattern emerges: Their positions relative to genes are highly variable, their sequences are extremely diverse, their activity in the cell is transient, they are weakly evolutionarily conserved, and their mechanisms of action are not fully understood. Even when a regulatory effect is measured, there is no consensus as to where exactly a regulatory element starts and ends along the genome. For this reason, currently available genome-wide annotations (6, 173)^.^ rely largely on indirect evidence of regulatory activity, although direct validation can be performed on selected sites (158). Recent experimental technologies suggest that it may soon be possible to measure regulatory effects on a large scale across the entire genome and across cell types (51, 52, 73, 156), shedding new light on the nature of regulatory elements.

### The Genome as a Frame of Reference for Scientific Communication

In effect,the human genome reference sequence is now more than a molecular measurement; itisa frame of reference that the biomedical community uses to connect its knowledge. For example, the HGNC gene symbols are used consistently from basic research to patient genetic reports. From an explosion of independent resources after the initial release of the human genome sequence, we are now observing a consolidation and standardization of the field, such that these resources will gradually form a consistent annotation of the sequence. After years of parallel work, the teams behind RefSeq and Ensembl/GENCODE are now cooperating within the Matched Annotation from NCBI and EMBL-EBI (MANE) project (43) to facilitate mappings from one system to the other. Similarly, Ensembl/GENCODE is collaborating with UniProt on the Gene Integration with Function,Taxonomy,and Sequence (GIFTS) resource (42),and UniProtis collaborating with the Protein Data Bank in Europe (PDBe) on the Structure Integration with Function, Taxonomy, and Sequence (SIFTS) resource (32).

Genomic variants,however,are currently referenced using multiple nomenclatures,which have positives and negatives. As early as 1993, a standard gene-based nomenclature was proposed (11) that would later become the Human Genome Variation Society notation. This approach, however, produces several ambiguous edge cases that hamper exact determination (68).

As the impact of genomics, and biology in general, has expanded to social and economic matters, greater attention has been paid to estimating and mitigating the consequences of sharing annotations. Whereas the academic field generally subscribes to open science to accelerate discovery—for example, in the Fort Lauderdale statement (165)—private companies and lawmakers have tended to prioritize data protection, for the sake of intellectual property as well as personal privacy [e.g., the Health Insurance Portability and Accountability Act in the United States (7) and the General Data Protection Regulation in the European Union (121)]. Respectful of the trust of human donors, the scientific community is currently developing secure methods to exchange knowledge and data without compromising individuals’ ethical and legal rights. Thus, the Global Alliance for Genomics and Health (16) is implementing software solutions such that data do not need to be copied across servers, let alone across territorial boundaries. Instead, computational analysis tasks will be distributed across data centers. Depending on the contractual and legal context, each analysis returns only summary statistics (which are not patient identifiable) or employs adequate encryption. To ensure the usefulness of this infrastructure work, efforts are ongoing to standardize the content available, for example, with respect to data quality or access rights.

## Conclusion

Nearly 20 years since its first public draft release, the annotated human genome sequence has reached adulthood and has become a mature reference that the scientific community, in both academia and industry, relies on intensively. In its initial years, many definitions had to be set, refined, and tested, and subsequent iterations led to highly variable annotations. However, under the stewardship of multiple organizations, it is gradually reaching stability, and it now offers a framework to support the consolidation of knowledge around gene sequences, gene regulatory networks, variants, population structure, and evolution.

Nevertheless, the genome sequence is far from retirement, as many of the novel uncharted aspects are regularly brought to light through better experimentation. While the list of protein-coding genes is converging to a fixed set, the definition of noncoding genes has yet to be settled. Regulatory elements and their interactions with genes are even more elusive. Genetic variation across the world’s population is not represented by today’s reference assembly, and the next major release will probably encode a collection of haplotypes. Finally, the human genome reference annotation community is now accountable for its ethical, legal, and societal impact on the world and is taking concrete steps to ensure that everyone benefits from the spectacular advances in the field.

## Figures and Tables

**Table 1:** Evidence relevant to the annotation of different types of genes

Biotype	Transcription data (INSDC, RNA-seq, PacBio, ONT)	Terminal transcription data (CAGE, RAMPAGE, polyA-seq)	Protein homology data (UniProt)	Protein experimental data (MS, ribo-seq)	Conservation data (PhyloCSF, PhastCons, GERP)	RNA secondary structure data (Infernal)	External expert database (miRBase, Rfam, IMGT)
Protein coding	Yes	Yes	Yes	Yes	Yes	No	No
lncRNA	Yes	Yes	No	No	No	No	No
sRNA	Yes	No	No	No	Yes	Yes	Yes
Pseudogene	No^a^	No^a^	Yes	No	Yes ^b^	No	No
IG/TR	No	No	No	Yes	No	No	Yes

This table illustrates the evidence types generally used by manual annotators in the Ensembl team to determine the correct structure and function of a transcript model. Protein-coding genes require transcriptomic evidence to define structure and terminal transcription data sets to define transcript start and end coordinates. Homology with UniProt and proteomics data informs or validates the decision to assign a transcript or locus the protein-coding biotype—that is, to decide whether a functional protein is encoded. Similarly, evolutionary conservation of sequence and of protein-coding potential also informs this decision. Decisions about protein-coding genes do not generally use RNA secondary structure or other expert databases, although they may be consulted on a case-by-case basis. The annotation of lncRNAs utilizes the same transcriptomic data sets as protein-coding genes; however, the absence of protein homology, experimental proteomics data, and conservation is a key determinant in choosing not to annotate a transcript as protein coding. For sRNAs, transcriptomic data sets, conservation data, RNA secondary structure data, and expert external databases are utilized. Pseudogenes are annotated based solely on their homology to annotated protein sequences, although transcriptomic data are used to support the transcribed pseudogene biotypes. IG/TR gene segments are annotated on the basis of protein experimental data and homology to IG/TR sequences from the IMGT database. Abbreviations: CAGE, cap analysis gene expression; GERP, Genomic Evolutionary Rate Profiling; IG, immunoglobulin; IMGT, International Immunogenetics; Infernal, Inference of RNA Alignment; INSDC, International Nucleotide Sequence Database Collaboration; lncRNA, long noncoding RNA; miRBase, MicroRNA Database; MS, mass spectrometry; ONT, Oxford Nanopore Technologies; PacBio, Pacific Biosciences; PhyloCSF, Phylogenetic Codon Substitution Frequencies; polyA-seq, polyA sequencing; RAMPAGE, RNA annotation and mapping of promoters for the analysis of gene expression; ribo-seq, ribosome profiling; RNA-seq, RNA sequencing; sRNA, small RNA; TR, T cell receptor.

a For nontranscribed pseudogenes only; transcribed pseudogenes may be supported by these data.

b While pseudogenes are not conserved over large evolutionary distances, known artifacts in the whole-genome alignments on which conservation detection is based permit their identification with care.

## First Surprise: How Many Protein-Coding Genes are there in the Human Genome?

Estimates of the number of human protein-coding genes made around the initiation of the Human Genome Project were as high as 100,000, although the numbers fell quite rapidly over the following decade and a half to reach equilibrium at around 20,000 (26, 46, 77), a figure that is frequently stated to this day. More recently, even lower counts of approximately 19,000 have been predicted (44), and it is this figure that more closely refects the number of functional protein-coding genes in the reference genome sequence found in the RefSeq and Ensembl/GENCODE catalogs, although other resources, such as CHESS, predict more (120). It might also surprise those making some of the higher predictions that the number of protein-coding genes in the human reference genome sequence is approximately 2,500 lower than the equivalent number for the mouse reference genome sequence produced by identical manual annotation workflows. However, despite the aspirations of the Human Genome Project and con-fdent predictions that a final protein-coding gene count is close, we do not yet have the definitive number (see Figure 4).

## Second Surprise: Lilliput Genes

One class of loci that suggests our current coding catalogs may be more incomplete than we believe are the small ORFs (smORFs), which are very short proteins that can still play an important functional role at the level of the cell or organism. While reference protein-coding gene catalogs do contain smORFs, including *STRIT1* (107), those that have been found either show very strong signals of conservation or purifying selection or have been comprehensively biochemically characterized, and there remains the possibility that (potentially a very large number of) smORFs with weaker signals of conservation remain unannotated.

One feature that is frequently described as a subclass of smORFs is upstream ORFs (uORFs), which lie in the 5′ untranslated regions of protein-coding loci. uORFs regulate the ability of the translating ribosome to access the translation initiation site of the primary coding sequence of a transcript (100). Again, there has been little functional characterization of uORFs, but where such data exist, they generally support the role of the uORFs as purely regulatory with the proteins they encode, although there are exceptions (137). Regulatory uORFs are not currently included in reference annotation sets even where they have been characterized.

## Third Surprise: Win Some, Lose Some

In a few rare cases, pseudogenes can evolve novel functionality. For example, transcripts originating from the *PTENP1* pseudogene of the functional human *PTEN* locus function as microRNA decoys, regulating the binding of microRNAs to the 3′ untranslated region of the parent locus (123). A prerequisite for novel gain of function is that a pseudogene be transcribed, and although there is an increasing volume of evidence for the transcription of both duplicated and retrotransposed pseudogenes, there is very little experimental evidence that gain of novel function can occur at the transcript level.

## Fourth Surprise: Coding Noncoding RNA?

By definition, lncRNAs do not encode functional proteins; however, their translation is more complex than this fact suggests. In experiments to identify evidence for translation, such as proteomics (mass spectrometry) and ribo-seq, signals of translation are frequently detected (71, 85, 166). Transcripts from lncRNAs clearly come into contact with ribosomes, and indeed, lncRNA loci have been demonstrated to be regulated via the act of translation and the NMD process that requires translation (147). Similarly, computational methods to identify regions of lncRNAs with protein-coding potential (e.g., from cross-species conservation) also find evidence that some lncRNAs could encode proteins (162). However, when subject to expert review, lncRNAs with a signal of protein-coding potential almost always fail to reach the standards required for reference annotation sets, with the signals explicable as technical or biological noise (168). Where a transcript or locus annotated as a lncRNA does pass the threshold for annotation as encodinga functional protein,both the transcript and locus are switchedinthe protein-coding biotype—that is, they are changed from being considered lncRNAs to being considered protein-coding genes, and as such, there is currently no recognition of protein-coding lncRNA genes. This may be challenged by the future discovery of protein-coding transcripts that possess demonstrable function at the transcript level. While the literature seems to suggest the existence of such loci, so far no examples have passed the threshold for inclusion in reference annotation sets.

## Fifth Surprise: Nonsense-Mediated Decay—Transcription’s Autocorrect

The NMD cellular mRNA surveillance pathway detects mRNAs with premature termination codons (PTCs) and promotes their degradation. NMD is a highly efficient mechanism for the cell to eliminate mRNAs, thus buffering the biological consequences of irregular splicing. While the precise mechanisms of NMD are complex and not fully understood, a PTC is defined as a stop codon that lies more than 50–55 base pairs upstream of a splice site, with an exon junction complex (EJC) deposited on it during the splicing process. If the EJC is fewer than 50 bases downstream of the PTC, the ribosome will have progressed sufficiently to displace the EJC from the mRNA, and NMD will not be initiated unless additional EJCs lie further downstream (103).

The function of NMD in degrading PTC-containing transcripts that are potentially damaging to the cell is well established, but a role in the active posttranscriptional regulation of genes has recently been identified. The first cases to be identified were genes encoding splicing factors of the SRSF family involved in the regulation of alternative splicing (90). These splicing factors were shown to autoregulate by directing their own splicing to produce SRSF2 transcripts sensitive to NMD when SRSF2 concentration is elevated. The functional link between alternative splicing and NMD provides a mechanism for fne-tuning gene expression, and this mechanism has been described as regulated unproductive splicing and translation (RUST) and alternative splicing coupled NMD (AS-NMD). Variants that affect these mechanisms have been shown to cause disease; for example, a variant in the PTC-containing exon of an NMD transcript in *SNRPB* was shown to dysregulate the splicing of the exon, causing cerebro-costo-mandibular syndrome (93), while variants promoting the inclusion of a poison exon in *SCN1A* cause Dravet syndrome (23).
